# A Young Female With Borderline Lepromatous Leprosy and Tuberculous Lymphadenitis: A Rare Coinfection

**DOI:** 10.7759/cureus.23892

**Published:** 2022-04-06

**Authors:** Chowdhury Adnan Sami, Shadman Shabab Hassan, Abed Hussain Khan, Md. Nazmul Hasan, Shohael Mahmud Arafat

**Affiliations:** 1 Department of Internal Medicine, Bangabandhu Sheikh Mujib Medical University, Dhaka, BGD

**Keywords:** severe neuropathy, coinfection, type 2 lepra reaction, leprosy, tuberculosis

## Abstract

In Bangladesh, tuberculosis and leprosy are endemic mycobacterial diseases; however, co-infection is rarely seen. Our patient had a high-grade fever, symmetrical polyarthritis, polymorphous erythematous lesions, widespread lymphadenopathy, peripheral neuropathy, bilaterally thickened ulnar nerves, and claw hands. A lymph node biopsy revealed tuberculosis having acid-fast bacilli with caseating epithelioid histiocytic granuloma. Cutaneous lesions and sural nerve biopsies indicated borderline lepromatous leprosy. Fite-Faraco stain showed the presence of lepra bacilli in the biopsied sural nerve. Mantoux test showed 15 mm induration in 72 hours. Nerve conduction study (NCS) showed severe sensory-motor polyneuropathy (axonal) of all four limbs. Prednisolone and thalidomide for severe type-2 lepra response and category-01 antituberculosis medication and multidrug therapy for multibacillary leprosy improved the patient's condition. Bacille Calmette-Guérin (BCG) vaccination in the community might protect against tuberculosis and leprosy, thus reducing such coinfection. However, reduced cell-mediated immunity might promote latent tuberculosis reactivation or super-infection in individuals with multi-bacilli illnesses.

## Introduction

Tuberculosis (TB), a multisystem disease caused by Mycobacterium tuberculosis, is still a major worldwide health issue. About 10.0 million got sick in 2019 alone, with mortality among HIV-negative and positive people being 1.2 and 0.208 million, respectively [1]. On the other hand, Mycobacterium leprae causes leprosy, damaging the skin and peripheral nerves. Globally, 202,189 new cases were detected in 2019 [2]. The clinical symptoms of both illnesses depend on the host's immunological response. Yet, it is unknown if co-infection protects against the other or if the host's immune response favors mycobacteriosis. Dual infections are linked with high mortality (37%) and morbidity (5.5%) [3]. Immunological leprosy responses emerge as lepra reactions before, during, or even years after therapy. Both are frequent in developing countries, yet even in endemic regions, co-infection is uncommon (0.02 per 100,000 population) [4]. Only a few case reports of TB and leprosy cohabitation in the same patient exist in the literature and are rarely documented in Bangladesh.

## Case presentation

**Figure 1:**
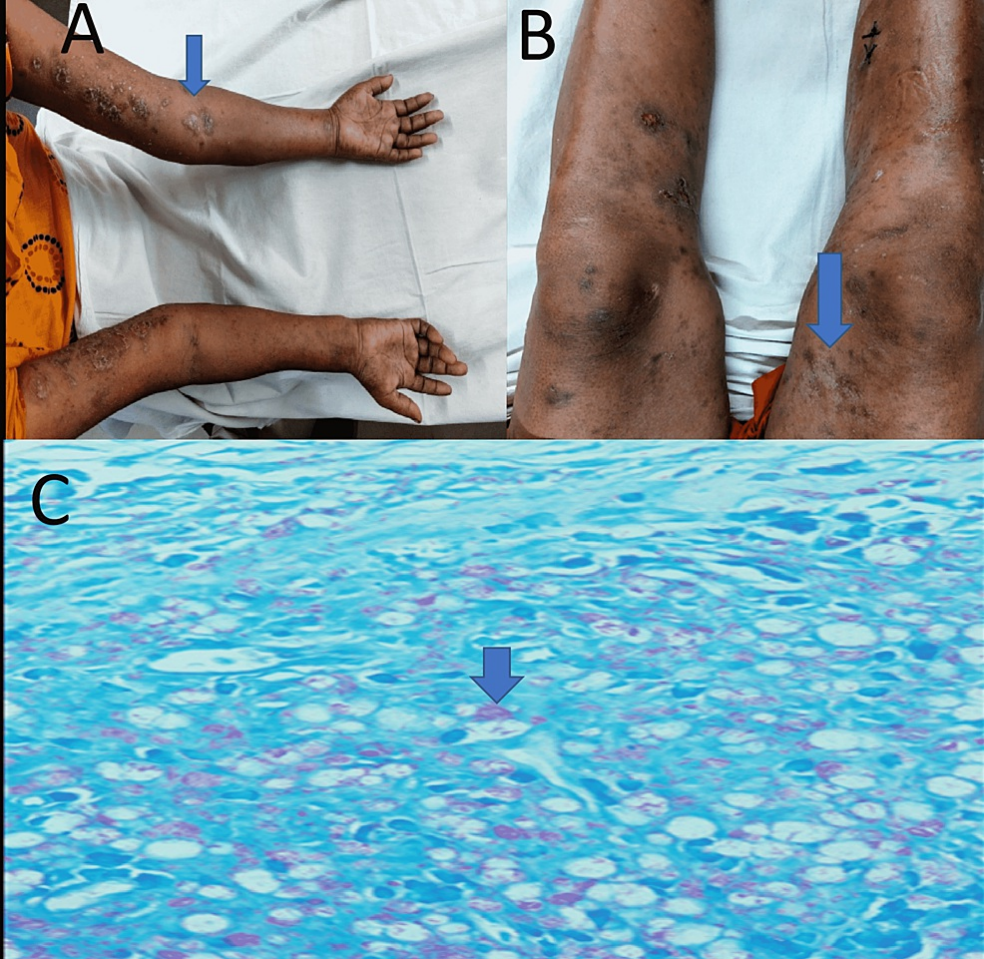
Polymorphous erythematous to violaceous lesions (arrows) in upper limb (A), lower limb (B), microscopic view of biopsied lymph node with Fite-Faraco stain showing globi of lepra bacilli (arrow) (C).

A 35-year-old previously Bacille Calmette-Guérin (BCG) vaccinated woman presented to us with a high-grade fever, generalized rash, followed by painful swelling of both ankles, wrist, and small joints of both hands for 1½ months. She also had a burning sensation in both hands and feet for one month. She had a history of substantial weight loss, approximately 8 kg during her illness. We found generalized lymphadenopathy; the nodes were firm, non-tender, and mobile with no discharging sinus. In addition, she had multiple non-tender polymorphous erythematous to violaceous lesions, including papules, plaques, nodules, and pustules; although sensation was intact over the lesions. Lesions were distributed symmetrically involving the post-auricular region, face, trunk, buttocks, and both upper and lower extremities (Figure 1A, 1B). There were also numerous post-inflammatory hyperpigmented lesions of various sizes with overlying scales. Examination of the upper limb revealed swelling of wrist joints and Grade-III tenderness in both wrists, metacarpophalangeal, proximal interphalangeal, elbow and shoulder joints with restricted active and passive movements due to pain. Examination of the lower limb also revealed swelling of both ankle joints and feet, Grade-III tenderness over the same region with painful restriction of active and passive movements. Nervous system examination features suggestive of predominant sensory neuropathy were present along the radial, median and ulnar nerve distribution in both upper limbs with bilaterally thickened ulnar nerves and the tibial and common fibular nerve distribution in both lower limbs. There was significant wasting of small muscles of both hands with dorsal guttering and complete clawing deformity of the left hand and partial clawing of the right hand. Although, vibration, proprioception and coordination were intact.

Laboratory investigations showed hemoglobin level of 7.2 gm/dl, erythrocyte sedimentation rate (ESR) 120 mm in 1st hour, total white blood cell (WBC) 16,000/mm³ with 81% neutrophils and 15% lymphocytes in complete blood count with C-reactive protein (CRP) was >200 mg/L. Peripheral blood film (PBF) showed microcytic hypochromic anemia with neutrophilic leukocytosis. Her anemia was attributed to iron deficiency anemia with transferrin saturation of 12%. Her liver function and renal function test, chest X-ray, and ultrasonography of the whole abdomen were normal. Histopathology of a biopsied inguinal lymph node supported the diagnosis of tuberculous lymphadenitis, with the presence of numerous Acid-Fast Bacilli on Ziehl-Neelsen stain with caseating epithelioid histiocytic granulomatous inflammation. To differentiate tuberculous bacilli with lepra bacilli we have done a genexpert of biopsied lymph node tissue, which was negative. But caseating epithelioid histiocytic granuloma is one of the cardinal features of tuberculosis infection rather than leprosy. Mantoux test showed 15 mm induration in 72 hours, which also favors the diagnosis of concomitant tuberculosis. Hansen's bacilli were seen in slit skin smears from skin lesions on the arm. Several globi of Acid-Fast Bacilli within macrophages on Fite-Faraco stain of biopsied inguinal lymph node led to the diagnosis of concurrent leprosy (Figure 1C). As there were several globi of lepra bacilli found in histopathology, which was suggestive of multibacillary disease, we did not perform any additional investigation to see the bacillary index. Additionally, borderline lepromatous leprosy was established from histopathology of biopsied leg skin, which revealed a moderate infiltration of foamy histiocytes, lymphocytes, polymorphs, and plasma cells in the dermis at the perivascular, periadnexal, and perilobular levels with numerous lepra bacilli forming globi were seen on Fite-Faraco stained sections.

**Figure 2:**
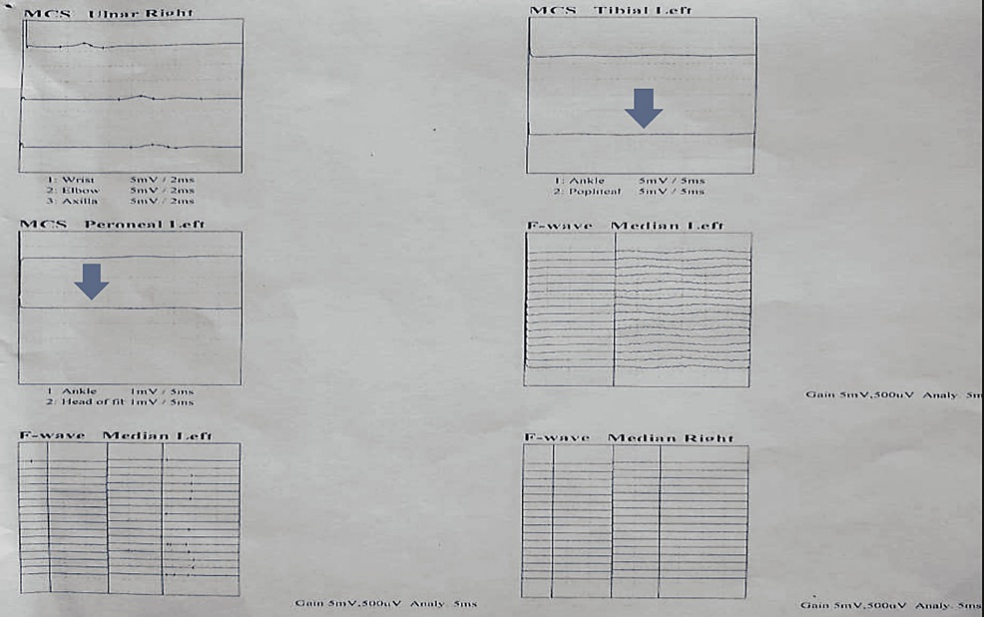
Nerve conduction study of upper and lower limbs with reduced amplitude (arrow).

Fite-Faraco staining of the biopsied sural nerve indicated the presence of lepra bacilli. Following that, this patient underwent a nerve conduction investigation, of which results are consistent with severe sensory-motor polyneuropathy (axonal) involving the upper and lower limbs (Figure 2).

For her tuberculous lymphadenitis, we administered a daily fixed-dose combination of Category 1 antitubercular medications combining isoniazid (75 mg), rifampicin (150 mg), pyrazinamide (400 mg), and ethambutol (275 mg). We also started a monthly loading dose of clofazimine (300 mg) followed by a daily dose of clofazimine (50 mg) and a daily dose of dapsone (100 mg). In addition, for her type 2 lepra reaction, a daily dose of prednisolone (40 mg) was started. Her iron deficiency was corrected with injectable ferric carboxymaltose. Her fever, rash, and swelling of both hands and feet gradually subsided, and she was discharged to home on request with advice about ongoing therapy.

Two months later, she again presented to us with features of severe type-2 lepra reactions including painful nodular lesions all over the body, suggestive of erythema nodosum leprosum and severe neuropathy. Hemoglobin level was 7.2 gm/dL, ESR was 115 mm in the first hour, total WBC count was 12,000/mm^3^, platelet count was 3,50,000/mm^3^, PBF revealed dimorphic anemia with neutrophilic leukocytosis, and CRP was 52 mg/L. The reticulocyte count was raised to 6.72x10^9^/L, lactate dehydrogenase (LDH) was 530 U/L, Coomb's tests (direct and indirect) were negative and serum bilirubin was also normal. She was diagnosed with relapsed type 2 lepra reaction with dapsone-induced hemolysis. The daily dapsone (100 mg) was stopped, and 400 mg of ofloxacin was added daily. For her lepra reaction relapse, she was again started on a daily dose of prednisolone 50 mg, and thalidomide was added as a daily dose of 200 mg. However, to reduce the risk of further neurological deterioration we prescribed thalidomide below the usual recommended dose, as the patient already had severe axonal peripheral neuropathy. Within six weeks, her rash, arthritis, and neuropathy markedly improved along with biochemical inflammatory markers.

## Discussion

Concurrent infection with Mycobacterium tuberculosis and Mycobacterium leprae is extremely rare in the recent literature, with new cases detected at a rate of about 0.02 instances per 100,000 populations each year [4]. For evaluation of a possible co-existence between the two illnesses, archaeological materials spanning from the Roman period to the twelfth century were analyzed using polymerase chain reaction (PCR) for Mycobacterium leprae and Mycobacterium tuberculosis DNA. Numerous specimens with paleopathological evidence of leprosy included DNA from both infections, demonstrating that both illnesses formerly coexisted [5].

The pathogenesis of dual infection has been proposed to be a defective innate immunity against both mycobacterial species; nevertheless, it appears that the energy in leprosy is pathogen-specific [6]. Additionally, it is suggested that impaired cell-mediated immunity contributes to the reactivation of latent tuberculosis in leprosy patients; lepromatous leprosy patients have a decreased TNF-alpha response and decreased inducible signaling molecules such as chemokine ligand-2 (CCL-2), resulting in unrestricted mycobacteria growth and dissemination [7].

Clinically and by the histopathological report, our patient was diagnosed with tuberculous lymphadenitis and borderline lepromatous leprosy (BLL) with type 2 lepra response. The presence of enlarged lymph nodes infiltrated by coalescent epithelioid histiocytic granulomas with central caseous necrosis is consistent with granulomas found in Mycobacterium tuberculosis infections [8] rather than with granulomas found in Mycobacterium leprae infections, which are characterized by the absence of caseous necrosis [9].

It has been reported that people with leprosy who are also afflicted with tuberculosis have a more severe case of tuberculosis [10]. However, in our case, tuberculosis manifested itself in a far more benign form, tuberculous lymphadenitis. Additionally, in recorded cases, most co-infected individuals developed pulmonary tuberculosis. Nonetheless, extrapulmonary tuberculosis was uncommon in the co-infection group (laryngeal TB) [11], central nervous system TB [4], and cutaneous tuberculosis [12]. Extrapulmonary tuberculosis occurred in our patient in the form of widespread lymphadenopathy.

Peripheral edema and severe inflammation are symptoms of Type 1 lepra responses. This is accomplished by the infiltration of interferon and tumor necrosis factor into the skin and nerves, where they secrete CD4-positive cells. Tender papules and nodules with indications of systemic toxicity - fever, malaise, and joint pain - identify type 2 lepra responses or erythema nodosum leprosum (ENL) in this case. Type 2 lepra responses are more likely to exhibit extensive skin infiltration, numerous nerve involvement, and impairment [13]. Sensory nerves were found to be the most damaged in nerve conduction studies. Patients with BLL may develop a silent progressive degenerative peripheral neuropathy that worsens despite long-term steroid treatment at high doses [14]. A rare pattern of peripheral nervous system involvement in leprosy is segmental necrotizing granulomatous neuritis in nerve biopsy specimens, which show epithelioid granulomas with caseous necrosis. Although, borderline tuberculoid (BT) and tuberculoid (TT) leprosy has been reported in this association [15]. However, our patient has histologically confirmed lepromatous leprosy, which does not manifest as caseation necrosis.

According to the WHO's treatment guidelines for ENL, a severe case of ENL should initially be treated with low prednisolone dosages. If the patient does not react to prednisolone, they are started on clofazimine alone or, in most instances, on a combination of clofazimine and prednisolone. When patients do not respond to either clofazimine or clofazimine and prednisolone, thalidomide is explored. Additionally, in the treatment of ENL, thalidomide should be administered in conjunction with steroids in a dosage range of 100 to 400 mg as a split dose in cases of neuritis [16]. Dapsone-induced hemolytic anemia, a frequent adverse drug reaction in leprosy patients taking daily dapsone dose, is often addressed by discontinuing the drug; however, in those cases, ofloxacin or minocycline may be given to patients as an alternate therapy [17].

## Conclusions

While tuberculosis and leprosy are both prevalent in endemic areas, co-infection with both mycobacteria is significantly less common in most leprosy-endemic nations. A possible explanation for the drop in reported instances of co-infection is the community's high prevalence of BCG vaccination, which has previously been shown to confer some protection against leprosy and tuberculosis. So, a high degree of clinical suspicion may detect such co-infections, lowering the total burden of death and morbidity.
